# Host-specific assemblages typify gut microbial communities of related insect species

**DOI:** 10.1186/2193-1801-3-138

**Published:** 2014-03-11

**Authors:** Zakee L Sabree, Nancy A Moran

**Affiliations:** Department of Ecology and Evolutionary Biology, Yale University, New Haven, CT 06511 USA; Department of Evolution, Ecology and Organismal Biology, The Ohio State University, Columbus, OH 43210 USA; Section of Integrative Biology, The University of Texas at Austin, Austin, TX 78712 USA

**Keywords:** Cockroaches, Termites, Gut microbiota, *Blattabacterium*

## Abstract

Mutualisms between microbes and insects are ubiquitous and facilitate exploitation of various trophic niches by host insects. Dictyopterans (mantids, cockroaches and termites) exhibit trophisms that range from omnivory to strict wood-feeding and maintain beneficial symbioses with the obligate endosymbiont, *Blattabacterium*, and/or diverse gut microbiomes that include cellulolytic and diazotrophic microbes. While *Blattabacterium* in omnivorous *Periplaneta* is fully capable of provisioning essential amino acids, in wood-feeding dictyopterans it has lost many genes for their biosynthesis (*Mastotermes* and *Cryptocercus*) or is completely absent (*Heterotermes*). The conspicuous functional degradation and absence of *Blattabacterium* in most strict wood-feeding dictyopteran insects suggest that alternative means of acquiring nutrients limited in their diet are being employed. A 16S rRNA gene amplicon resequencing approach was used to deeply sample the composition and diversity of gut communities in related dictyopteran insects to explore the possibility of shifts in symbiont allegiances during termite and cockroach evolution. The gut microbiome of *Periplaneta*, which has a fully functional *Blattabacterium*, exhibited the greatest within-sample operational taxonomic unit (OTU) diversity and abundance variability than those of *Mastotermes* and *Cryptocercus*, whose *Blattabacterium* have shrunken genomes and reduced nutrient provisioning capabilities. *Heterotermes* lacks *Blattabacterium* and a single OTU that was 95% identical to a *Bacteroidia*-assigned diazotrophic endosymbiont of an anaerobic cellulolytic protist termite gut inhabitant samples consistently dominates its gut microbiome. Many host-specific OTUs were identified in all host genera, some of which had not been previously detected, indicating that deep sampling by pyrotag sequencing has revealed new taxa that remain to be functionally characterized. Further analysis is required to uncover how consistently detected taxa in the cockroach and termite gut microbiomes, as well as the total community, contribute to host diet choice and impact the fate of *Blattabacterium* in dictyopterans.

**Electronic supplementary material:**

The online version of this article (doi:10.1186/2193-1801-3-138) contains supplementary material, which is available to authorized users.

## Background

Successful collaborations between microbes and their arthropod hosts have enabled exploitation of a wide range of trophic niches. Maternally inherited intracellular bacteria and/or gut-inhabiting microbial assemblages involved in host nutrition are found in insects that persist on specialized (e.g. plant juices, animal blood, wood or decaying plant material) or omnivorous diets (Moran et al. 2008; Hongoh 2010). Cockroaches (Dictyoptera: Blattaria) are host to both an obligate intracellular symbiont and a species-rich gut microbial community, while most termites (Dictyoptera: Isoptera) host only the latter (Lo et al. 2003; Hongoh 2010; Schauer et al. 2012). Nearly all cockroaches harbor the intracellular mutualist, *Blattabacterium sp.* (heretofore referred to as *Blattabacterium*), which has codiversified with their insect hosts over millions of years and in most cases they can recycle nitrogen from ammonia and urea metabolic wastes into the production of essential and nonessential amino acids (Sabree et al. 2009). Termites share a common ancestor with the wood roach *Cryptocercus spp.* (heretofore referred to as *Cryptocercus*) via *Mastotermes darwiniensis* (heretofore referred to as *Mastotermes*), which forms the basal branch in termite phylogenies, and as such these species represent transitional stages between cockroaches and termites. When compared to their modern cockroach relatives, termites sport several striking distinctions that include advanced social behaviors, dramatic physiological modifications (e.g. enlarged hindguts, perpetual neoteny, thin cuticles) (Nalepa 2011) and, with *Mastotermes* as the only known exception, loss of the heritable *Blattabacterium* symbiont (Bell et al. 2007). *Cryptocercus* and *Mastotermes*, also exhibit many of these characteristics and retain *Blattabacterium* (Neef et al. 2011; Sabree et al. 2012). It is surprising that the genomes of *Blattabacterium* in these two host insects lack genes involved in the biosynthesis of some essential amino acids, given that their hosts are limited for nitrogen given that they thrive on wood-based diets typically low in protein (0.03-0.7% nitrogen (Merrill & Cowling 1966; Tayasu et al. 1994)). Many intracellular bacterial mutualists of phytophagous insects with highly reduced genomes still retain essential amino acid biosynthesis pathway presumably to supplement their low nitrogen diet (Sabree et al. 2013). Thus, the functional deterioration of *Blattabacterium* in *Cryptocercus* and *M. darwiniensis* and its complete elimination in other termites suggest alternative means of obtaining and retaining nitrogen in these insects.

Emergent mutualisms with gut microbes that can provide the same nutrients as the ancient endosymbiont while conferring new functions may have been a context for functional deterioration and eventual loss of *Blattabacterium* in *Cryptocercus* and *Mastotermes* and termites, respectively. Supporting evidence in this regard would be the discovery of nitrogen fixation in termites (Benemann 1973), its association with hindgut bacteria (Yamada et al. 2007; Potrikus & Breznak 1977; Kudo et al. 1998; Ohkuma et al. 1999), and identification of genes underlying nitrogen fixation and essential amino acid biosynthesis in hindgut bacteria in various termite species (Wertz et al. 2012; Isanapong et al. 2012). Additionally, ‘lower’ termites and *Cryptocercus* are aided in their trophic specialization on wood by lignocellulosic hindgut microbes that include both bacteria (Hongoh 2010; Mattéotti et al. 2011; Abt et al. 2012) and protists (Tartar et al. 2009; Scharf et al. 2011; Carpenter et al. 2011; Tamschick & Radek 2013). These protists are themselves hosts to intra- and extra-cellular bacterial symbionts (Hongoh et al. 2008a; Hongoh et al. 2008b; Desai & Brune 2012; Strassert et al. 2012). Since *Blattabacterium* can neither degrade cellulose nor fix nitrogen, acquisition of organisms capable of these functions enables their host to exploit an abundant dietary substrate and represents a significant improvement on resource utilization afforded by *Blattabacterium*.

A 16S rRNA gene amplicon resequencing approach (Schloss & Handelsman 2003; Tringe & Hugenholtz 2008) was used to deeply sample the composition and diversity of gut communities in dictyopteran insects to explore the possibility of shifts in symbiont allegiances during the evolution of termites from cockroaches. We expected that if *Cryptocercus* and termites are reliant upon their gut microbiomes in the context of a functionally diminished or absent *Blattabacterium*, respectively, then their community profiles will be stable across intraspecific individuals and abundant bacterial operational taxonomic unit (OTU) therein would be consistently detected. Additionally, metabolic profiles and available genomic information from taxa related to abundant gut microbiome members detected in this study were used to make general inferences about their possible roles in host trophic ecology.

## Results and discussion

### Sampling effort, community diversity analysis and general taxonomic profiling

The gut microbiota of two termite and two cockroach species were surveyed by pyrosequencing to identify and quantify community membership, and to compare intra- and inter-host community profiles in the context of 1) the host’s diet, 2) presence or absence of *Blattabacterium* and 3) the inferred functional capacity of *Blattabacterium*. A total of 276,850 high-quality pyrotags representative of 16S rRNA gene V6-V9 region amplicons were clustered into 1,152 OTUs and included in subsequent analyses (Additional file 1:Table S1). Observed and estimated OTU richness and rarefaction analyses suggest that near-comprehensive sampling of the termite and *Cryptocercus* microbiota could be achieved in fewer than 35,000 pyrotags, but this may not be sufficient for the *Periplaneta* microbiota due to the relatively high OTU richness therein (Table 1; Additional file 2: Figure S1).

**Table 1:** **Insect gut community alpha diversity**

Sample	Pyrotags	Observed OTUs	Species richness	OTU_obs_/OTU_ACE_	Species evenness	Average distance^1^
Ha01	33427	189	191.19 (+/− 4.5)	0.99	2.50 (+/− 0.04)	
Ha02	30441	150	171.85 (+/− 17.0)	0.87	1.18 (+/− 0.01)	Ha: 0.23
Ha03	34266	191	199.90 (+/− 9.6)	0.96	1.85 (+/− 0.02)	
Md01	16610	287	294.43 (+/− 7.7)	0.97	18.44 (+/− 0.6)	
Md02	14964	303	316.55 (+/− 11.1)	0.96	35.33 (+/− 1.0)	Md: 0.38
Md03	14086	254	268.44 (+/− 11.6)	0.95	20.41 (+/− 0.5)	
Cp01	12476	319	329.65 (+/− 9.4)	0.97	39.65 (+/− 1.6)	
Cp02	14482	321	338.55 (+/− 12.6)	0.95	24.35 (+/− 1.0)	Cp: 0.31
Cp03	12504	334	341.44 (+/− 7.5)	0.98	34.71 (+/− 1.4)	
PaW01	20767	275	314.39 (+/− 22.7)	0.87	16.08 (+/− 0.4)	
PaW02	2081	225	282.93 (+/− 29.9)	0.80	32.38 (+/− 4.2)	PaW: 0.57
PaW03	30565	391	401.13 (+/− 9.2)	0.97	26.39 (+/− 0.7)	
PaL01	4208	299	342.61 (+/− 23.2)	0.87	54.68 (+/− 4.5)	
PaL02	11801	364	380.07 (+/− 11.6)	0.96	44.60 (+/− 2.0)	PaL: 0.60
PaL03	24172	384	400.78 (+/− 12.3)	0.96	60.96 (+/− 1.3)	

Species richness was estimated using the abundance coverage estimator (ACE) and evenness was demonstrated as the inverse of Simpson’s estimation of evenness. 95% confidence indicators are noted in parenthesis for both measures. Ha-*Heterotermes aureus*, Md-*Mastotermes darwiniensis*, Cp-*Cryptocercus punctulatus,* PaW-*Periplaneta americana* wild-caught, PaL-*P. americana* lab-reared.

1-Average within-group sample distances were calculated using Multi-Response Permutation Procedures using the Bray-Curtis distance measure. Test statistic: −8.719, observed delta: 0.4142, expected delta: 0.8829, chance-corrected within group agreement: 0.5309, P-value: 7.0e-8.

**Figure 1:**
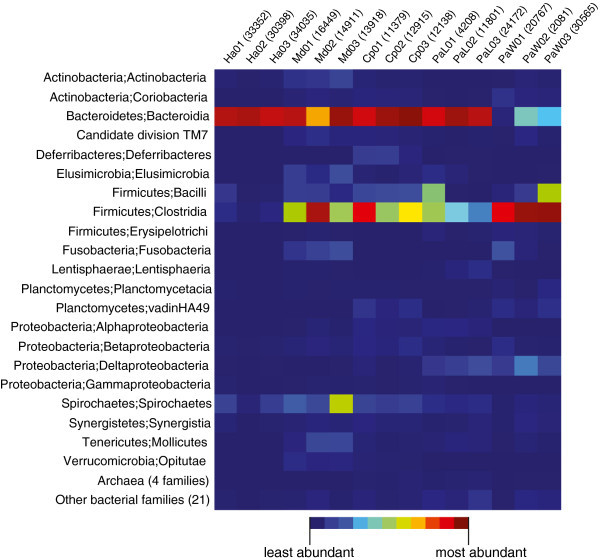
***Bacteroidia*** **and** ***Clostridia*** **predominate in most cockroach and termite gut microbiota.** Heatmap depicts the relative abundances of pyrotags assigned to each taxonomic class; total number of pyrotags representative of each sample are parenthesized.

Sequences representative of each of the 1,152 OTUs were taxonomically classified by comparing them to SILVA (version 108;) and nr (accessed May 6, 2012) databases using blastn. OTUs were assigned to over 24 bacterial phyla that include *Fusobacteria*, *Deferribacteres*, *Cyanobacteria*, *Verrucomicrobia*, *Elusimicrobia*, TM7, *Planctomycetes*, *Tenericutes*, *Synergistetes*, *Actinobacteria*, *Spirochaetes*, *Proteobacteria*, *Bacteroidetes*, and *Firmicutes* (Figure 1; Table 2). Single OTUs assigned to the *Bacteroidia* and *Clostridia* classes were abundant (defined as >1%) in every sample, and, when combined, predominant in all four host species (Additional file 3: Table S2). *Bacilli*, *Elusimicrobia*, *Deltaproteobacteria* and *Spirochaetes* OTUs were detected, but they were not uniformly abundant, in every sample. With some exceptions, OTUs assigned to many of the remaining families were not abundant (i.e. <1%) and/or were absent from some samples or sample groups.

**Table 2:** **Relative abundance of bacterial families present within insect gut communities**

Taxonomic designation	Ha01	Ha02	Ha03	Md01	Md02	Md03	Cp01	Cp02	Cp03	PaL01	PaL02	PaL03	PaW01	PaW02	PaW03
*Actinobacteria; Actinobacteria*	1.06 (8)	0.23 (7)	1.01 (8)	2.74 (8)	2.74 (10)	4.14 (9)	0.70 (10)	0.37 (10)	0.49 (9)	n.d. (0)	n.d. (0)	n.d. (1)	0.18 (6)	0.91 (7)	0.51 (8)
*Actinobacteria; Coriobacteria*	0.05 (2)	0.02 (2)	0.01 (2)	0.55 (3)	1.21 (3)	0.47 (3)	1.00 (4)	1.07 (4)	0.86 (4)	0.12 (2)	0.08 (2)	0.13 (2)	4.49 (2)	1.44 (2)	0.99 (2)
*Bacteroidetes; Bacteroidia*	75.83 (21)	95.68 (17)	83.53 (18)	42.41 (52)	28.31 (51)	33.95 (50)	36.66 (46)	48.57 (50)	44.71 (50)	41.02 (89)	58.21 (88)	56.59 (100)	1.51 (50)	19.75 (55)	14.83 (88)
Candidate division TM7	0.04 (2)	0.01 (1)	0.04 (2)	1.53 (13)	2.22 (14)	0.94 (11)	1.08 (12)	1.09 (10)	0.65 (8)	0.26 (4)	0.09 (3)	0.31 (6)	0.31 (5)	1.87 (7)	0.29 (6)
*Deferribacteres; Deferribacteres*	n.d. (0)	n.d. (0)	n.d. (0)	n.d. (0)	n.d. (0)	n.d. (0)	3.51 (1)	4.81 (1)	1.45 (1)	0.02 (1)	0.23 (2)	0.23 (2)	n.d. (0)	n.d. (0)	0.05 (2)
*Elusimicrobia; Elusimicrobia*	0.04 (2)	0.01 (2)	0.02 (2)	4.31 (8)	1.87 (9)	6.07 (7)	0.57 (2)	0.26 (3)	0.8 (2)	2.16 (5)	1.07 (5)	0.26 (3)	0.01 (1)	0.05 (1)	0.1 (3)
*Firmicutes; Bacilli*	5.92 (9)	0.2 (7)	0.81 (11)	4.18 (5)	3.47 (5)	1.54 (4)	5.79 (9)	7.88 (8)	7.98 (8)	20.29 (7)	0.24 (5)	0.01 (2)	1.54 (8)	4.13 (6)	24.8 (10)
*Firmicutes; Clostridia*	4.00 (67)	1.05 (50)	3.11 (67)	24.75 (97)	38.2 (109)	17.7 (84)	35.02 (140)	24.5 (138)	29.19 (147)	22.98 (117)	22.4 (151)	15.86 (151)	61.81 (142)	48.82 (100)	44.06 (186)
*Firmicutes; Erysipelotrichi*	n.d. (0)	n.d. (0)	n.d. (0)	0.16 (2)	0.43 (2)	0.07 (2)	0.05 (1)	0.09 (1)	0.02 (1)	0.9 (6)	0.23 (7)	0.55 (7)	1.58 (5)	0.96 (3)	0.4 (5)
*Fusobacteria; Fusobacteria*	n.d. (0)	n.d. (0)	n.d. (0)	3.00 (1)	4.25 (1)	6.12 (1)	n.d. (0)	n.d. (0)	n.d. (0)	0.02 (1)	n.d. (0)	n.d. (0)	13.71 (1)	1.11 (1)	0.22 (1)
*Lentisphaerae; Lentisphaeria*	n.d. (0)	n.d. (0)	n.d. (0)	0.30 (3)	0.25 (3)	0.1 (2)	n.d. (0)	n.d. (0)	n.d. (0)	0.26 (4)	1.49 (5)	3.17 (5)	0.18 (2)	n.d. (0)	0.08 (2)
*Planctomycetes; Planctomycetacia*	0.43 (2)	0.01 (1)	0.13 (2)	0.24 (3)	0.12 (2)	0.19 (3)	0.24 (1)	0.26 (2)	0.12 (2)	0.12 (3)	0.29 (6)	0.14 (5)	0.83 (5)	0.19 (4)	1.85 (6)
*Planctomycetes;* vadinHA49	0.04 (1)	0.03 (1)	0.11 (1)	n.d. (0)	n.d. (0)	n.d. (0)	2.65 (4)	1.49 (3)	2.36 (4)	0.05 (2)	0.28 (5)	0.67 (5)	3.29 (4)	1.06 (4)	2.65 (5)
*Proteobacteria; Alphaproteobacteria*	0.08 (4)	0.06 (3)	0.04 (4)	0.14 (6)	0.61 (9)	0.24 (4)	1.62 (15)	1.17 (14)	0.86 (14)	1.88 (9)	2.3 (14)	1.42 (16)	0.03 (3)	0.1 (1)	0.04 (5)
*Proteobacteria; Betaproteobacteria*	0.70 (5)	0.42 (6)	0.71 (6)	0.73 (5)	1.22 (5)	0.6 (4)	1.52 (7)	1.36 (9)	2.39 (9)	0.71 (3)	0.36 (6)	0.19 (6)	3.39 (6)	0.43 (1)	0.27 (3)
*Proteobacteria; Deltaproteobacteria*	0.35 (5)	0.06 (5)	0.41 (6)	0.02 (1)	0.03 (1)	0.15 (1)	0.85 (9)	0.16 (7)	0.46 (8)	3.26 (20)	5.94 (26)	10.38 (32)	5.84 (20)	12.88 (15)	5.69 (26)
*Proteobacteria; Gammaproteobacteria*	0.59 (2)	0.01 (1)	0.1 (2)	0.22 (2)	0.34 (3)	0.23 (2)	0.35 (4)	0.19 (6)	0.42 (6)	0.64 (2)	0.18 (1)	n.d. (0)	0.03 (2)	0.05 (1)	0.04 (2)
*Spirochaetes; Spirochaetes*	8.84 (39)	1.87 (32)	8.34 (40)	9.67 (45)	5.93 (39)	20.02 (42)	4.74 (15)	4.47 (15)	4.88 (16)	2.38 (7)	2.69 (11)	2.96 (12)	0.43 (1)	1.3 (4)	0.72 (6)
*Synergistetes; Synergistia*	1.24 (5)	0.12 (5)	0.75 (5)	0.55 (7)	1.09 (8)	0.6 (7)	1.31 (5)	0.77 (5)	0.79 (5)	0.26 (2)	0.47 (4)	1.21 (4)	0.35 (3)	1.78 (2)	0.81 (3)
*Tenericutes; Mollicutes*	0.01 (1)	0.02 (1)	0.02 (1)	1.42 (7)	5.02 (7)	4.87 (5)	0.66 (7)	0.33 (6)	0.47 (8)	0.64 (4)	1.55 (4)	1.25 (5)	0.22 (3)	0.86 (3)	0.25 (5)
*Verrucomicrobia; Opitutae*	0.02 (1)	0.01 (1)	0.03 (1)	2.30 (5)	1.3 (4)	1.27 (5)	0.55 (6)	0.53 (7)	0.31 (8)	0.07 (1)	0.08 (2)	0.05 (2)	n.d. (0)	0.05 (1)	0.04 (1)
Other bacterial families (21 families)	0.77 (8)	0.19 (5)	0.83 (8)	0.52 (10)	1.14 (14)	0.19 (5)	0.98 (12)	0.38 (12)	0.61 (14)	1.69 (9)	1.2 (14)	3.88 (16)	0.2 (4)	2.07 (5)	1.22 (12)
*Archaea* (4 families)	n.d. (0)	n.d. (0)	n.d. (0)	0.25 (3)	0.25 (3)	0.54 (2)	0.14 (2)	0.26 (3)	0.17 (3)	0.26 (1)	0.64 (3)	0.73 (2)	0.06 (2)	0.19 (2)	0.1 (4)

Values represent the percentage of all taxonomically-assigned OTUs within each sample. Parenthesized values represent the real number of OTUs within each sample assigned to that taxonomic group. n.d.-not detected.

### Predominant taxa are host specific and consistently abundant

**Figure 2:**
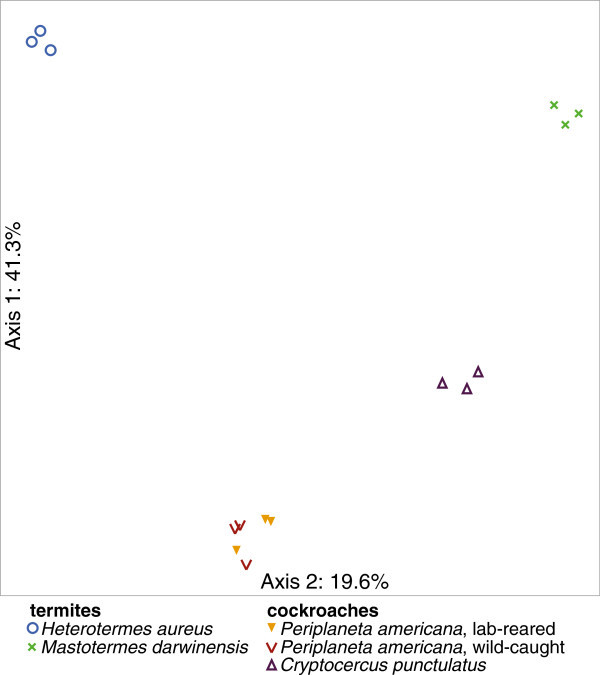
**Insect community samples form host-defined clusters.** Nonmetric multidimensional scaling was used to visualize community diversity and relatedness. A randomly subsampled, size-normalized (n = 2,050) pyrotag abundance data table was used.

OTUs assigned to the *Bacteroidia* predominate in nearly all host gut communities but few were shared between cockroaches and termites (Table 2). Similarly, *Clostridia* were abundant in every host, but to lesser degree (representing <4% of the total OTUs) in *H. aureus*. Like the *Bacteroidia*-assigned OTUs, none were shared across both termites and cockroaches. Given that 59-96% of the pyrotags in each sample could be assigned to either class, yet few OTUs were shared, the observed host-defined sample clustering in the NMS analysis is not unexpected (Figure 2). It is not surprising to find members of both classes in all of the gut microbiomes of hosts in this study as cultivated bacteroidia and clostridia 1) range from aerotolerant to strict anaerobes, exhibit a wide range of potentially host-beneficial carbon fermentative metabolisms, including cellulose degradation by various clostridial genera (Tracy et al. 2012), 2) are well-adapted to the dynamic gut environment and 3) are common amongst the gut flora of healthy invertebrates and vertebrates (Schleifer 2009; Engel & Moran 2013).

OTUs assigned to the *Tenericutes* and *Elusimicrobia* were abundant only in *Mastotermes.* Members of the *Tenericutes*, namely *Phytoplasma*, *Mycoplasma* and *Spiroplasma* are well-adapted to reside in various animal and plant hosts largely as pathogens (Garnier et al. 2001; Gasparich 2010) but a recent report of *Spiroplasma* conferring protection against parasitic nematodes in some *Drosophila* species (Haselkorn et al. 2013) indicates other interactions between tenericutes and their eukaryotic hosts are possible.

Members of the *Elusimicrobia* were initially detected in *Reticulitermes speratus* hindguts (Ohkuma & Kudo 1996; Hongoh et al. 2003) and have since been detected in the guts of various phytophagous insects and terrestrial habitats (Herlemann et al. 2007). *Elusimicrobium minutum* was the first cultivated member of this clade (Geissinger et al. 2009) and this strict anaerobe generates ATP through typical fermentative pathways and generates some amino acids but likely relies upon other microbiome members, the host or the host’s diet for additional required nutrients. In contrast, production of many essential amino acids using ammonia by an *Elusimicrobia* endosymbiont of a cellulolytic protist inhabitant of *R. speratus* was inferred from an analysis of its genome and they are accessible to the host via digestion of the protist or by uncharacterized transport mechanisms (Hongoh et al. 2008a).

Although *Spirochaetes* have been well documented in termite gut communities (Paster & Dewhirst 2000; Rosenthal et al. 2011; Köhler et al. 2012), no single OTU was abundant in either termite host species examined. Hydrogen metabolism has been proposed as one of the functions of spirochaetes in termite guts (Rosenthal et al. 2011) but further characterization is necessary. Combined OTUs assigned to the *Spirochaetes* were present and >1% of the sequence reads in all insects except two wild-caught *P. americana* samples, yet only three occurrences were noted where single OTUs were >1% of the sequence reads for a sample (Additional file 3: Table S2). Since the OTUs were defined at ≥95% sequence identity, the *Spirochaetes* detected in the termites exhibited greater within-sample diversity and evenness of abundance than that observed for OTUs assigned to the *Bacteroidia* or *Clostridia* classes in the same termites.

### Protist-associated OTUs predominate the microbiota of insects lacking fully functional *Blattabacterium* endosymbionts

Nearly all cockroaches harbor the obligate intracellular mutualist, *Blattabacterium*, and its absence in all termites except *Mastotermes* presents an opportunity to explore the possibility of mutualist replacement within the dictyopterans. We hypothesized that loss of *Blattabacterium* could only be tolerated if newly acquired microbes 1) were abundant in the gut (a proxy for being well-adapted to the host environment and an important member of the community), 2) could be reliably transmitted to offspring and 3) capable of supplying nutrients required by the host. Results from this survey of the microbiota indicate that microbiota samples from *Heterotermes,* which lacks *Blattabacterium*, had the least amount of OTU diversity and evenness and exhibited the least community profile variability, as indicated by the low average distance (Table 1). Conversely, microbiota samples from *P. americana*, which harbors *Blattabacterium* with all of its essential amino acid biosynthesis pathways intact, were more variable for relative abundance, OTU richness, evenness and thus exhibited the greatest distance between samples from the same host. Average distances for the microbiota samples from *Cryptocercus* and *Mastotermes*, whose *Blattabacterium* associates are deprived of many of the aforementioned biosynthetic pathways, were intermediate to those of *Heterotermes* and *Periplaneta*.

Accounting for a large proportion of the *Heterotermes* microbiota is a single *Bacteroidia*-assigned OTU (OTU_0035) that had a best hit (95% identity) to a nitrogen-fixing (diazotrophic) endosymbiont of the cellulolytic trichonymphid protist, *Pseudotrichonympha grassii*, that resides in *Coptotermes formosanus* hindguts (Hongoh et al. 2008b) (Additional file 3: Table S2). The prevalence of OTU_0035 in the *H. aureus* samples suggests that it may be an essential member of the community, which would be reasonable if it too is diazotrophic and partnered with a cellulolytic protist. Soldiers and young instars obtain nutrients and gut microbes from the hindgut fluids of conspecifics via proctodeal trophallaxis (Machida et al. 2001; Nalepa et al. 2001). This practice would ensure the reliable communication of the gut microbiota that include oxygen-sensitive microbes, some of whom can fix nitrogen, a process that is inactivated by oxygen. The tight mutualism between the N_2_-fixing endosymbiont and the cellulolytic *P. grasii* combines functions essential for the termite to thrive on plant-based carbon sources in a single trophic mutualism. If the role of *Blattabacterium* is to provision amino acids and vitamins missing in the host’s diet, its role would be tangential in the presence of the cellulolytic protist-diazotrophic bacteria symbiosis because the N_2_-fixing protist endosymbiont can generate the same assortment of nutrients and *Blattabacterium* cannot degrade cellulose or fix atmospheric nitrogen.

Both *Mastotermes* and *Cryptocercus* have cellulose-based diets, exhibit trophallaxic behavior and still retain *Blattabacterium,* albeit with reduced genomes and diminished nutrient provisioning abilities. Unlike *Blattabacterium* in other cockroaches, which are capable of making all ten essential amino acids, the endosymbiont in *Mastotermes* and *Cryptocercus* encode the genes for the production of only five or six essential amino acids, respectively (Neef et al. 2011; Sabree et al. 2012). In most cases members of the *Bacteroidia* were the most abundant in *Cryptocercus* and *Mastotermes*, but no single OTU was as abundant as OTU_0035 in *Heterotermes*.

Two abundant, protist-associated bacteroidial OTUs (OTU_1551 and OTU_0214) in *Mastotermes* were only 93% identical to OTU_0035, which suggests little gene flow between these bacteroidial taxa and isolation of their protist hosts within their respective termite hosts would support this. The devescovinid *Mixotricha paradoxa* is abundant in *Mastotermes* but the functions of its associated bacteroidia remain to be determined. Additional OTUs detected in *Mastotermes* that were related to bacterial symbionts of gut protists were taxonomically assigned to the *Elusimicrobia and Tenericutes*. Protists are abundant symbiotic inhabitants of termites (Ohkuma & Brune 2011; Desai & Brune 2012), which likely contributes to the relative abundance of their associated bacteria. The low percent identity to available taxon-assigned sequences suggests that this is the first published detection of the *Tenericutes*-assigned gut protist symbiont in *M. darwiniensis*. It is possible that the *M. darwiniensis Elusimicrobia* detected in this study plays a role in life of its host that is analogous to that of *R. speratus* protist symbionts given that their hosts have similar diets.

Many of the *Bacteroidia and Clostridia*-assigned OTUs in the *P. americana* gut communities had best hits to amplicons obtained from either *Shelfordella lateralis* (Schauer et al. 2012) or various termites. *S. lateralis* and *P. americana* are both part of the Blattidae (Blattoidea) family, which suggests that the shared OTUs may represent taxa that are well-adapted to their cockroach hosts and were acquired following divergence of the *Shelfordella-Periplaneta* (Blattoidea) and *Cryptocercus* (Blaberoidea) clades. Deep sampling of gut microfloral diversity of other host taxa in both of these superfamilies is necessary to test this hypothesis. The role of taxa shared by *Periplaneta* and *Shelfordella* in cockroach host development and/or trophisms remain to be characterized. Finally, the relative abundance of pyrotags representing the abundant OTUs assigned to the *Bacteroidia* and *Clostridia* classes varied significantly (T-test: p < 0.0003) between the samples obtained from wild-caught and lab-reared *P. americana*. This supports the hypothesis that *P. americana* harbors some host-specific bacteria, as evidenced by the ordination analyses, but diet and/or habitat can impact their prevalence.

### Ammonia-oxidizing and sulfate-reducing bacteria in cockroaches

OTUs assigned to the *Planctomycetes* and *Deltaproteobacteria* were largely shared by and unique to the cockroaches. Known physiological characteristics of *Planctomycetes* members are the lack of peptidoglycan in their cell walls, intracellular compartmentalization, sterol biosynthesis, and budding reproduction (for review (Fuerst & Sagulenko 2011)). Additionally, so-called ‘annamox’ members are chemoautotrophic anaerobes capable of oxidizing ammonia to dinitrogen and are being successfully exploited for energy-efficient removal of nitrogenous wastes in wastewater treatment (Siegrist et al. 2008; Shi et al. 2013). Ammonia comprises much of the nitrogenous wastes externally excreted by cockroaches (Cochran 1985) and annamox *Planctomycetes* present in their hindguts may utilize this surplus to generate ATP via the annamoxosome (van Niftrik & Jetten 2012) and, by effect, may have a detoxifying effect by reducing the concentration of ammonia present in the hindgut.

Deltaproteobacteria-assigned OTUs in *Cryptocercus* and *Periplaneta* hindguts had best hits to amplicons that were assigned to the *Desulfobacteriaceae* and *Desulfovibrionaceae* families. Many cultivated members of both groups are strict anaerobes residing in marine sediments that are capable of coupling sulfate-reduction and energy production (Muyzer & Stams 2008). Additionally, Candidatus “Desulfovibrio trychonymphae”, an endosymbiont of an anaerobic *R. speratus-*inhabiting protist, has also been shown to have and express genes involved in sulfate-reduction (Sato et al. 2009), but it is not clear if this is their primary function in the hindgut microbiome.

## Conclusions

The gut microbiomes of dictyopteran insects surveyed in this study are comprised of many bacteria from the same class-level taxonomic groups (e.g. *Bacteroidia, Clostridia*, *Spirochaetes* and *Bacilli*) but distinct sub-lineages were observed when OTUs were resolved at the ≥95% sequence identity cutoff, indicating the presence of many relatively abundant host-specific taxa. The gut communities of the strict wood-feeding insects had bacterial taxa known to be associated with cellulolytic protists and fewer shared OTUs while the OTU diversity was generally greater and more variable in the omnivorous *P. americana.* If the abundant endosymbiont of the *H. aureus* cellulolytic trichonymphid protist is diazotrophic and capable of provisioning amino acids to its protist host that is itself digested by the termite, then acquisition of this and other gut bacterial-protist or bacterial symbioses that facilitated wood-feeding could have contributed to the shift in nutritional reliance from *Blattabacterium* to the gut microbiome. *M. darwiniensis* and *Cryptocercus* have microbiomes that are more diverse than that of *H. aureus*, harbor cellulolytic protists with bacterial symbionts and also sport many of the host physiological and behavioral modifications observed in *H. aureus.* Given that *M. darwiniensis* and *Cryptocercus* are sister taxa, both harbor *Blattabacterium spp.* that are functionally reduced and *M. darwiniensis* is basal to termites, it is possible that these host gut microbiomes represent intermediate stages of a more stable community that is essential for wood-feeding. Unlike in *M. darwiniensis* and *Cryptocercus*, the *P. americana Blattabacterium* is equipped to provision vitamins and a near-complete suite of amino acids to its host, which may reduce its reliance upon the gut microbiome for these functions. Elevated average distances between within-group samples for *P. americana* gut microbiota membership suggest this possibility but the presence of shared OTUs between the wild-caught and lab-reared *P. americana* indicates a host-specific microbiota that is present regardless of diet or lifestyle. The functions of these members remain to be determined.

Deep sequencing has helped to identify a number of previously undetected taxa in this study, and others seeking to profile cockroach and termite microbiomes (Köhler et al. 2012; Schauer et al. 2012; Huang et al. 2013; Boucias et al. 2013) that likely represent novel strains, species or genera, indicating that the guts of dictyopteran insect, of which there are about 8,500 species, contain a wealth of novel bacterial diversity. It is clear that further functional characterization of abundant cockroach and termite gut microbiome members is necessary and will likely reveal some new biological activities.

## Methods

### DNA extraction and multiplexed sample preparation

Entire guts were dissected from fresh or ethanol-preserved specimens from the following sources: *Heterotermes aureus* (Tucson, Arizona, USA; July 2008), *Mastotermes darwiniensis* (Marlow Lagoon, Northern Territory, Australia), *Cryptocercus punctulatus* (Mountain Lake, Virginia, USA), wild-caught *Periplaneta americana* (near the University of Arizona gymnasium, Tucson, Arizona, USA, July 2010, 33°C and 25% relative humidity), lab-reared *P. americana* (lab colony fed on dog food containing 28% amino acids, provided water *ad libatum* and maintained at 24C and 35% relative humidity). Three adult individuals captured from each habitat were sampled. DNA was prepared using the Power Soil DNA Isolation Kit (MoBio, San Diego, USA) according to the supplied protocol. 50 ng of template DNA was used in PCR amplifications performed in triplicate in 30 μL reactions containing 0.4 μM bacteria-specific forward primer (TAXX-926 F: 5′-{adapter}-{barcode}-AAACTYAAAKGAATTGACGG-3′; (Lane 1991; Engelbrektson et al. 2010)) that were uniquely barcoded for multiplexing samples, 0.4 μM nonbarcoded universal primer (TB-1392R: 5′-{adapter}-TACGGYTACCTTGTTACGACTT-3′; (Ferris et al. 1996; Engelbrektson et al. 2010)) (see Additional file 4: Materials for primer sequences), 0.55 U Phusion Taq DNA polymerase (New England Biolabs, Massachusetts, USA), and 1 mM dNTP mix (Promega, Wisconsin, USA). Selected primers were used to amplify the hypervariable V6-V9 regions of the 16S rRNA gene (Sogin et al. 2006; Roesch et al. 2007). Reactions were initially denatured at 98°C for 1 min, followed by 25 cycles of 98°C for 10 sec, 55°C for 10 sec and 72°C for 15 sec and a final, single cycle of 72°C for 10 min. Amplification was confirmed by gel electrophoresis, and PCR products from amplifications performed in triplicate were pooled, purified using AmPure magnetic beads (Beckman Coulter, Indianapolis, USA) and quantified using the Qubit fluorometer (Life Technologies, New York, USA). Barcoded PCR products were combined, at a final concentration of 0.45 ng per reaction, into a single, multiplexed sample that was submitted to the University of Arizona Genomics Center for pyrosequencing on a Roche 454 FLX-Titanium system.

### Pyrotag processing and analysis

402,054 raw, barcoded amplicon sequences (“pyrotags”) were obtained from the pyrosequencing run. These were processed within the CLC Genomics Workbench (http://www.clcbio.com) to trim low-quality regions (<27 Phred score) from each pyrotag and to remove reads that were less than 400 bp in length and/or had one or more errors in the forward primer or barcode regions. MOTHUR was used for further pyrotag processing (version 1.24, (Schloss et al. 2009)) (for details, see Additional file 4: Materials), and pyrotags having ≥95% identity were clustered into operational taxonomic units (OTUs) in MOTHUR (cluster.split, method = furthest). OTUs that did not have at least two pyrotags present in at least two samples were excluded from our dataset to minimize the impact of possible contaminants and extremely rare OTUs on our analyses. OTUs were taxonomically classified by blastn-based (BLAST+, version 2.2.26; parameters -task blastn -outfmt 6 -evalue 1e-50; (Camacho et al. 2009)) searches of Silva (version 108, (Pruesse et al. 2007)) and NCBI ‘nt’ nucleotide databases with representative sequences of each OTU. Acceptable alignments included only those for which >85% of the query and ‘hit’ (or ‘subject’ in BLAST+ parlance) sequences were aligned. Available taxonomic information for the top hits were used to define OTUs. OTUs having hits to plastids or mitochondria were removed from the analysis. We used the abundance-based coverage estimator (ACE) (Chao et al. 1993) to conservatively predict the number of OTUs in each sample (richness) and the inverse Simpson diversity index (Simpson 1949) to estimate OTU evenness. Prior to our comparative community analyses, we generated a size-standardized subset of the original data that, in terms of the relative abundances of OTUs in each sample, was not significantly different (paired t-test, p > 0.05) from the original dataset by randomly sampling pyrotags from each OTU to 2,050 per sample for all samples. The subsampled dataset was used as input to test and visualize within-group and between-group differences using Multi-Response Permutation Procedures (MRPP) (Mielke 1984) and Nonmetric Multidimensional Scaling (NMS) (Kruskal 1964) within the PC-ORD (version 4.25) software package (McCune & Mefford 1999). The Bray-Curtis distance measure was used in both comparative analyses and a combination of low stress and maximum stability was sought for the NMS solution.

### Sequencing data accession number

Representative OTU names and corresponding NCBI GenBank accession numbers can be found in Additional file 4.

## Electronic supplementary material

Additional file 1: Table S1: Pyrotag processing data. Numbers indicate quantities of reads before and after trimming low quality base calls and removal of undersized reads. a-percentage of total remaining reads. b-percentage of total remaining OTUs. (DOCX 48 KB)

Additional file 2: Figure S1: Insect community sampling analysis. Rarefaction curves reflect sampling-without-replacement. A: Ha-*Heterotermes aureus*, B: Md-*Mastotermes darwiniensis*, C: PaW-*Periplaneta americana* wild-caught, D: PaL-*P. americana* lab-reared, E: Cp-*Cryptocercus punctulatus*. CI- 95% confidence intervals. Number of pyrotags are indicated on the x-axis and number of OTUs are indicated on the y-axis. (PDF 95 KB)

Additional file 3: Table S2: Best GenBank matches to abundant OTUs. OTUs that were present in all three samples for at least one host sample group and represented >1% of the total pyrotags in at least two samples per sample group are described. Asterisks indicate that fewer than 1% of the total pyrotags for that sample clustered with the corresponding OTU. Sequences representative of selected OTUs were compared to a local ‘nt’ database using blastn (parameters: −task blastn -outfmt 6) and sequences with the greatest ‘max identity’ and ‘query coverage’ were reported. All hits were to uncultured bacteria detected by PCR unless otherwise noted. Description information was obtained from the GenBank entry ‘definition’ and ‘accession’ entries. Accession numbers for best hits are parenthesized. Habitat information was obtained from the GenBank entry ‘source’ section.%ID- percent identity. ^- According to searches of ‘nt’ and ‘silva’ databases, GenBank entry AM422253.1 is incorrectly annotated as an “Uncultured Eubacterium sp”. and would not be a member of the Firmicutes. &- Best blast hit was to a bacterial isolate. (XLSX 40 KB)

Additional file 4: **Representative OTUs.** Text document includes representative OTU names and corresponding NCBI GenBank accession numbers. (TXT 21 KB)
