# Supplementary material for: Host-specific assemblages typify gut microbial communities of related insect species
Source: Springerplus. 2014 Mar 11;3:138. doi: 10.1186/2193-1801-3-138 (PMC3979980; doi:10.1186/2193-1801-3-138)
Supplement: Supplementary file 1 — Additional file 1: Table S1: Pyrotag processing data. Numbers indicate quantities of reads before and after trimming low quality base calls and removal of undersized reads. a-percentage of total remaining reads. b-percentage of total remaining OTUs. (DOCX 48 KB) [file 40064_2014_880_MOESM1_ESM.docx]

|  | **Pre-frequency filtering** | **Post-frequency filtering** |
| --- | --- | --- |
| **Average read length, bp** | 486 | 486 |
| **Total high-quality reads** | 292,790 | 276,850 (94.56%)^a^ |
| **OTUs, pre-frequency filter** | 6,360 | 1,152(18.11%)^b^ |
| **Average reads per sample** | 19,519 (range: 2,259-34,775) | 18,457 (range: 2,081-34,266) |

**Table S1. Pyrotag processing data**

Numbers indicate quantities of reads before and after trimming low quality base calls and removal of undersized reads.

a-percentage of total remaining reads

b-percentage of total remaining OTUs
