# Supplementary figures and images for: Host-specific assemblages typify gut microbial communities of related insect species
Source: Springerplus. 2014 Mar 11;3:138. doi: 10.1186/2193-1801-3-138 (PMC3979980; doi:10.1186/2193-1801-3-138)

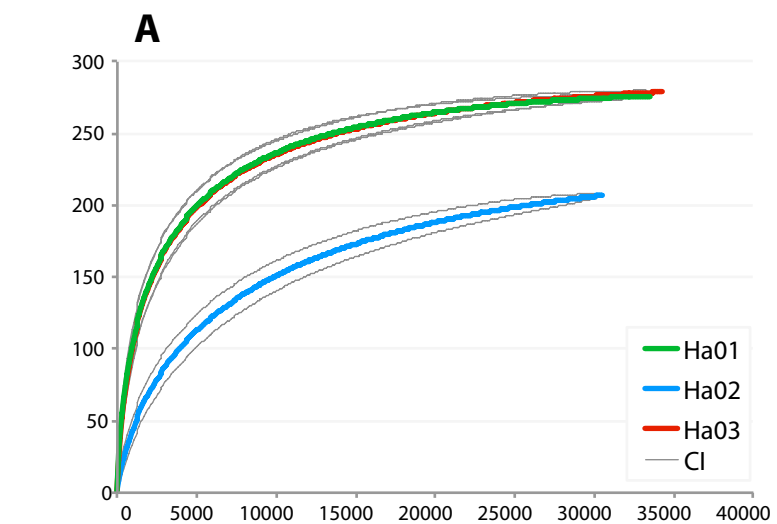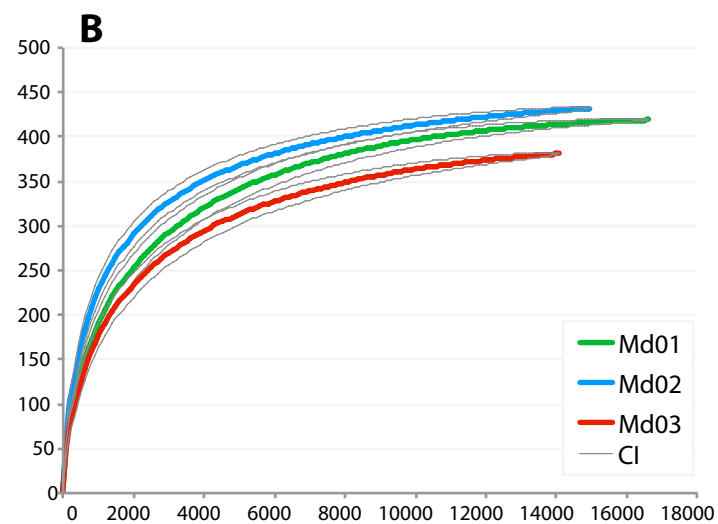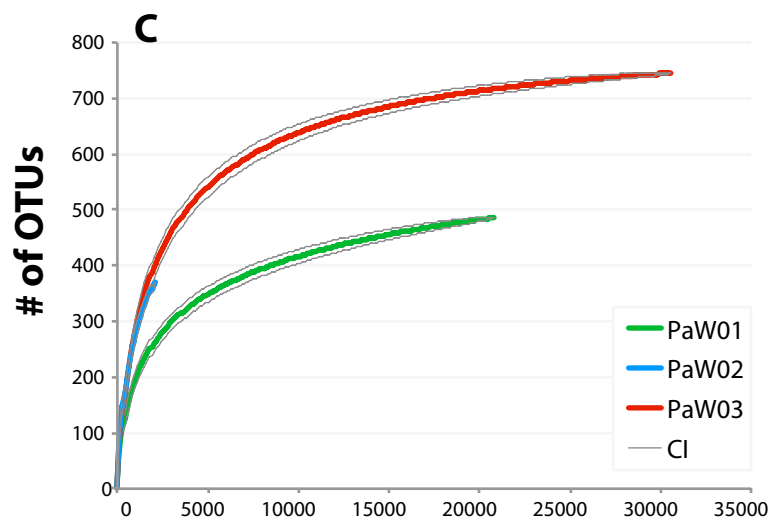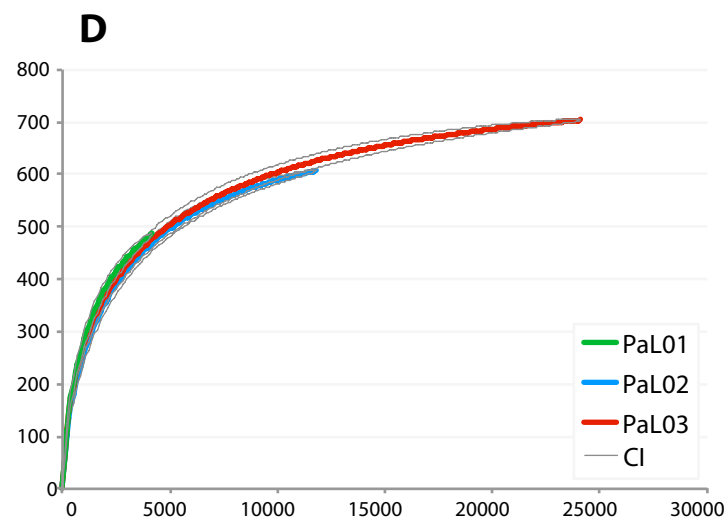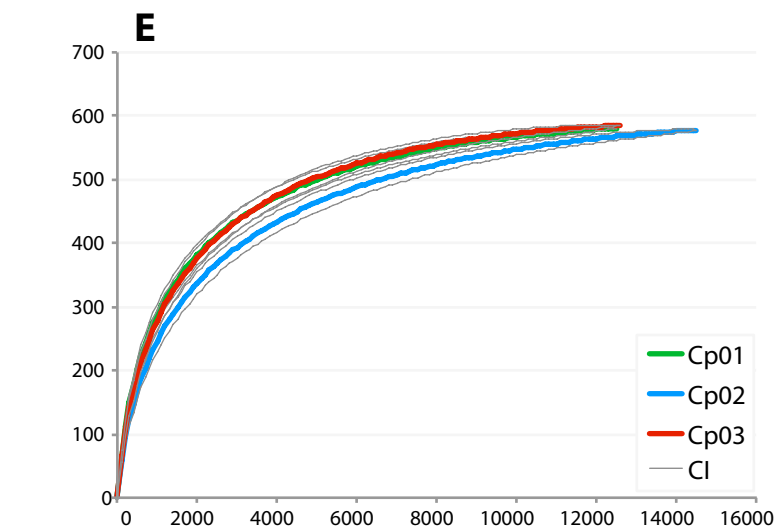

# of pyrotags

Supplement: Supplementary file 2 — Additional file 2: Figure S1: Insect community sampling analysis. Rarefaction curves reflect sampling-without-replacement. A: Ha-Heterotermes aureus, B: Md-Mastotermes darwiniensis, C: PaW-Periplaneta americana wild-caught, D: PaL-P. americana lab-reared, E: Cp-Cryptocercus punctulatus. CI- 95% confidence intervals. Number of pyrotags are indicated on the x-axis and number of OTUs are indicated on the y-axis. (PDF 95 KB) [file 40064_2014_880_MOESM2_ESM.pdf]
